# Brief guide to detecting ferroptosis

**DOI:** 10.1016/j.mocell.2025.100276

**Published:** 2025-09-10

**Authors:** Thu-Hang Thi Nghiem, Fedho Kusuma, Jeongmin Park, Yeonsoo Joe, Hun Taeg Chung, Jaeseok Han

**Affiliations:** 1 Department of Biological Sciences, University of Ulsan, Ulsan 44610, Republic of Korea; 2 Department of Integrated Biomedical Science, Soonchunhyang University, Cheonan 31151, Republic of Korea; 3 Natural Material Development Research Institute, Daegu Haany University, Gyeongsan 38610, Republic of Korea; 4 College of Korean Medicine, Daegu Haany University, Gyeongsan 38610, Republic of Korea; 5 Soonchunhyang Institute of Medi-bio Science (SIMS), Soonchunhyang University, Cheonan 31151, Republic of Korea

**Keywords:** Ferroptosis, Glutathione peroxidase 4, Iron metabolism, Lipid peroxidation, Multiparametric detection

## Abstract

Ferroptosis is an iron-dependent, lipid-peroxidation-driven form of regulated cell death that is distinct from apoptosis and necroptosis. Its involvement in various diseases highlights the need for reliable detection strategies. We provide a concise guide for ferroptosis detection, outlining key mechanisms, including iron metabolism, lipid remodeling, and antioxidant failure. Cellular, biochemical, genetic, and morphological methods, including viability assays, lipid reactive oxygen species probes, and electron microscopy, have been used to identify ferroptosis in vitro and in vivo. A multiparametric approach is emphasized to ensure the specificity and reproducibility.

## INTRODUCTION

Ferroptosis is a distinct form of regulated cell death characterized by the accumulation of lipid peroxides, setting it apart from apoptosis, necroptosis, and other cell death pathways. Since its initial characterization, ferroptosis has been implicated in a wide array of pathological conditions, including cancer, neurodegenerative diseases, ischemia–reperfusion injury, and metabolic disorders, underscoring its relevance across various biological systems (Feng et al., 2023). Given its emerging significance in disease pathogenesis and therapeutic response, the precise detection of ferroptosis is essential for both mechanistic investigation and translational application. This MiniResource provides a concise yet comprehensive overview of ferroptosis and outlines the key experimental approaches currently available for detecting this process in cellular and tissue contexts.

## DEFINITION AND HALLMARKS OF FERROPTOSIS

Ferroptosis is an iron-dependent and oxidative form of non–apoptotic cell death that plays a critical role in both physiological processes and pathological conditions (Lee and Dixon, 2025). It is distinguished from apoptosis, necroptosis, and autophagy based on unique morphological, biochemical, genetic, and pharmacological characteristics. The key hallmarks include iron accumulation, lipid peroxidation, transcriptional upregulation of acyl-CoA synthetase long-chain family member 4 (ACSL4) and other lipid-metabolizing enzymes, glutathione peroxidase 4 (GPX4) inactivation, and glutathione (GSH) depletion, which leads to oxidative membrane damage. Mitochondrial shrinkage with reduced cristae and preserved nuclear morphology are the characteristic ultrastructural features of ferroptosis (Dixon et al., 2012). Ferroptosis is triggered by agents such as erastin and RSL3 and rescued by inhibitors such as ferrostatin-1 and liproxstatin-1. These features form the foundation for detection strategies that combine biochemical assays, morphological analyses, gene expression profiling, and pharmacological validation.

## MOLECULAR MECHANISMS AND KEY REGULATORS

**Fig. 1:**
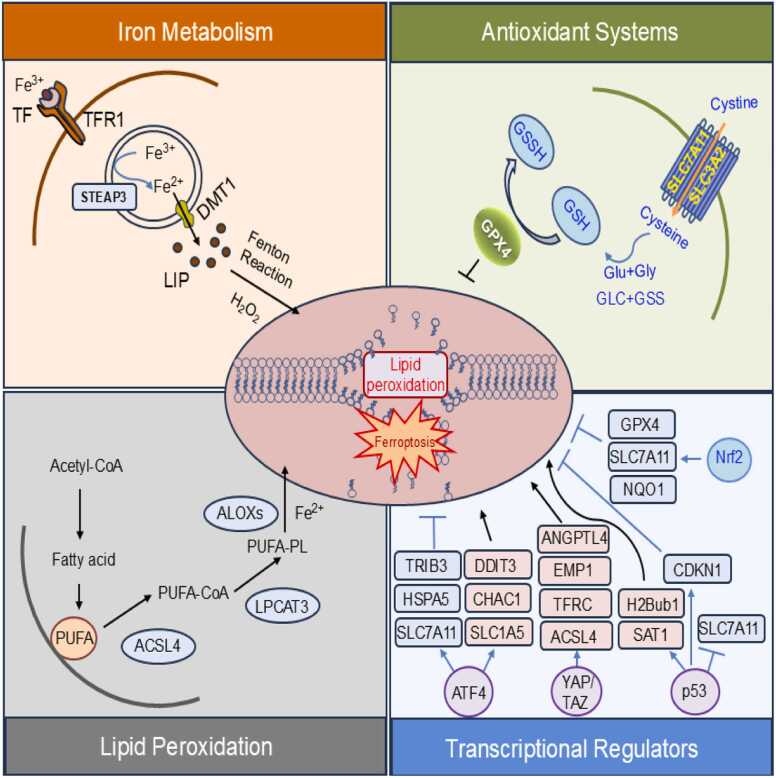
Key molecular pathways regulating ferroptosis. Ferroptosis is an iron-dependent form of regulated cell death driven by the accumulation of lipid peroxides. The schematic illustrates the major molecular pathways involved, including iron metabolism (orange), antioxidant systems (green), lipid peroxidation (gray), and transcriptional regulation (blue). TF: Transferrin, TFR1: Transferrin Receptor 1, STEAP3: Six-Transmembrane Epithelial Antigen of Prostate 3 (ferrireductase), DMT1: Divalent Metal Transporter 1LIP: Labile Iron Pool, Acetyl-CoA: Acetyl-Coenzyme A, PUFA: Polyunsaturated Fatty Acid, PUFA-PL: Polyunsaturated Fatty Acid–Phospholipid, PUFA-CoA: Polyunsaturated Fatty Acyl-Coenzyme A, LPCAT3: Lysophosphatidylcholine Acyltransferase 3, ACSL4: Acyl-CoA Synthetase Long-Chain Family Member 4, ALOXs: Arachidonate Lipoxygenases, GSH: Glutathione, GSSG: Glutathione Disulfide (oxidized GSH), GCL: Glutamate-Cysteine Ligase, GSS: Glutathione Synthetase, GPX4: Glutathione Peroxidase 4, SLC7A11: Solute Carrier Family 7 Member 11 (xCT), Nrf2: Nuclear Factor Erythroid 2–Related Factor 2, ATF4: Activating Transcription Factor 4, YAP/TAZ: Yes-Associated Protein / Transcriptional co-Activator with PDZ-binding motif, TRIB3: Tribbles Pseudokinase 3, DDIT3 (CHOP): DNA Damage-Inducible Transcript 3, HSPA5 (GRP78): Heat Shock Protein Family A Member 5, CHAC1: ChaC Glutathione-Specific Gamma-Glutamylcyclotransferase 1, SLC1A5: Solute Carrier Family 1 Member 5 (ASCT2), ANGPTL4: Angiopoietin-like 4, EMP1: Epithelial Membrane Protein 1, CDKN1A (p21): Cyclin-Dependent Kinase Inhibitor 1A, H2Bub1: Monoubiquitinated Histone H2B, SAT1: Spermidine/Spermine N1-Acetyltransferase 1.

Ferroptosis is regulated by a complex interplay between molecular pathways that coordinate iron homeostasis, lipid peroxidation, antioxidant systems, and transcriptional control (Chen et al., 2021). This section outlines the core mechanisms and key regulators of ferroptosis (Fig. 1).

### Iron Metabolism

Iron homeostasis is central to ferroptosis, as ferrous iron (Fe²⁺) drives lipid peroxidation via the Fenton reaction (Chen et al., 2020, Gao et al., 2015, Gumilar et al., 2023). Cells acquire iron through the transferrin-transferrin receptor 1 (TfR1) pathway, while STEAP3 and DMT1 facilitate Fe²⁺ import into the cytosol (Gao et al., 2015, Gumilar et al., 2023). Excess iron is stored in ferritin; however, nuclear receptor coactivator 4–mediated ferritinophagy releases Fe²⁺ into the labile iron pool (LIP), promoting ferroptosis (Hou et al., 2016). Ferroptotic sensitivity is further enhanced by ferroportin downregulation or hepcidin upregulation, both of which contribute to intracellular iron accumulation (Davaanyam et al., 2023, Geng et al., 2018, Ru et al., 2024, Zhang et al., 2021).

### Lipid Metabolism

Lipid remodeling is central to ferroptosis, as it governs the formation and accumulation of peroxidized polyunsaturated fatty acids (PUFAs) within membrane phospholipids (Lee et al., 2021, Suda et al., 2024). ACSL4 activates free PUFAs such as arachidonic acid or adrenic acid into acyl-CoA derivatives. These derivatives are then incorporated into phospholipids by lysophosphatidylcholine acyltransferase 3, forming PUFA-phosphatidylethanolamines. PUFA-phosphatidylethanolamines are highly susceptible to oxidation by lipoxygenases (eg, ALOX15) or non–enzymatic reactive oxygen species (ROS) generated via iron. The resulting lipid hydroperoxides (L-OOH) compromise membrane integrity if not detoxified by GPX4 (Lee et al., 2021).

### Antioxidant Systems

Antioxidant pathways are essential for preventing ferroptosis by detoxifying L-OOHs and maintaining redox balance. The central protective mechanism is the GPX4-GSH axis, in which GPX4 reduces L-OOH to lipid alcohols using GSH as a cofactor. GSH is synthesized from cysteine, which is imported via the system Xc^−^. Inhibition of system Xc^−^ or cystine deprivation leads to GSH depletion, promoting ferroptosis (Kuang et al., 2020). In addition, GPX4-independent pathways, such as the ferroptosis suppressor protein 1-CoQ10 (Doll et al., 2019) and GTP cyclohydrolase 1-tetrahydrobiopterin axis (Kraft et al., 2020), also suppress lipid peroxidation and confer resistance to ferroptosis (Ma et al., 2022).

### Transcriptional Regulators

Key transcription factors modulate ferroptosis by regulating genes involved in iron homeostasis, lipid peroxidation, and antioxidant defense (Tang et al., 2021). p53 promotes ferroptosis by repressing solute carrier family 7 member 11 (SLC7A11), limiting cystine uptake and GSH synthesis (Jiang et al., 2015), although it may also exert context–dependent inhibitory effects (Tarangelo et al., 2018, Xie et al., 2017). NFE2L2, also known as NRF2, generally protects against ferroptosis by upregulating genes, including GPX4, SLC7A11, ferritin, and NAD(P)H quinone dehydrogenase 1 (NQO1) (Anandhan et al., 2023, Xiang et al., 2024). In contrast, YAP/TAZ enhances ferroptosis by inducing ACSL4 and transferrin receptor, promoting PUFA incorporation and iron uptake (Xiang et al., 2022). ATF4, a central effector of the integrated stress response, also sensitizes cells to ferroptosis by upregulating ChaC GSH–specific gamma-glutamylcyclotransferase 1, which degrades GSH, and DNA damage inducible transcript 4, which inhibits mTORC1, and contextually regulating SLC7A11 (Dong et al., 2025, Nghiem et al., 2025). These transcriptional programs dynamically fine-tune ferroptotic sensitivity in response to cellular stress and metabolic state.

## EXPERIMENTAL APPROACHES TO DETECT FERROPTOSIS

### Cellular and Biochemical Assays

Cellular and biochemical assays are essential to detect ferroptosis by targeting its core features of iron accumulation, lipid peroxidation, GSH depletion, and GPX4 inactivation. These assays enable the functional evaluation of cell viability, oxidative stress, and redox imbalance using fluorescent probes, biochemical markers, and pharmacological modulators. Collectively, these results provide a foundational approach for confirming ferroptotic cell death in experimental systems.

#### Detection of Ferroptotic Cell Death

To assess ferroptotic cell death, viability assays are typically conducted using ferroptosis inducers such as erastin (a system Xc^−^ inhibitor) and RSL3 (a GPX4 inhibitor), alongside ferroptosis inhibitors such as ferrostatin-1 and liproxstatin-1, which act as radical-trapping antioxidants to suppress lipid peroxidation–induced death, confirming the ferroptotic nature of the process (Miotto et al., 2020, Yang et al., 2014). The comparative viability under these conditions provides key functional evidence, as summarized in Table 1 (Chen et al., 2024).

**Table 1:** Common assays for assessing ferroptotic cell death

Assay	Principle	Detection method	λ (nm)	Change	Reference	Cell types	System
CCK8	Reduction of WST-8 to formazan by viable cells	Microplate reader	450	↓	Zhang et al. (2024)	HT22, HepG2, PC12	In vitro
MTT	Mitochondrial reduction of MTT to formazan crystals	Microplate reader	570	↓	Ding et al. (2021), Kumar et al. (2018b)	A549, MCF-7, HEK293	In vitro
LDH	LDH release from damaged cell membranes	Microplate reader	490	↑	Du et al. (2023), Kumar et al. (2018a)	RAW264.7, HT1080, PC12	In vitro
SYTOX Green	DNA staining in membrane-compromised cells	Fluorescence microscope, flow cytometry	523	↑	Pei et al. (2022)	Mouse lung epithelial cells	In vitro
PI	DNA intercalation in dead cells with damaged membranes	Fluorescence microscope, flow cytometry	617	↑	Crowley et al. (2016), Tschuck et al. (2024)	Jurkat, HT29, primary neurons	In vitro

λ, detection wavelength; ↓, decreased signal; ↑, increased signal.

#### Detection of Lipid Peroxidation

As a central biochemical hallmark of ferroptosis (Chen et al., 2021), lipid peroxidation is typically assessed in live cells using oxidation–sensitive fluorescent probes, such as BODIPY 581/591 C11 and Liperfluo, enabling the semiquantitative analysis of lipid ROS via flow cytometry or fluorescence microscopy (Zeng et al., 2023). Complementary biochemical assays, including malondialdehyde quantification and 4-hydroxynonenal immunostaining, detected stable lipid peroxidation by-products in cultured cells and tissue sections. In addition, lipidomics-based profiling via LC-MS/MS offers high-resolution identification of oxidized phospholipid species, providing molecular specificity in complex biological samples or in vivo contexts. Collectively, these approaches provide a multilayered validation of the oxidative component of ferroptosis and are most effective when integrated with pharmacological or genetic modulation as summarized in Table 2.

**Table 2:** Common methods for assessing lipid peroxidation in ferroptosis studies

Category	Assay	Principle	Detection	λ / Mode	Change	Reference	Cell/tissue	System
Fluorescence	BODIPY 581/591 C11	Lipophilic dye shifts from red to green upon oxidation by lipid ROS	Fluorescence microscopy, flow cytometry	581/591 → 510 nm (Ex/Em: 488/510 nm)	↑	Drummen et al. (2002), Magtanong et al. (2022)	HT1080, HepG2, fibroblasts	In vitro
	Liperfluo	Probe detects lipid hydroperoxides; emits green fluorescence after reaction	Fluorescence microscopy, flow cytometry	488/530 nm	↑	Dai et al. (2023), Saimoto et al. (2025)	AML12, neuronal cells	In vitro
ELISA	4-HNE	Competitive ELISA using anti–4-HNE antibody to detect lipid aldehyde adducts	Microplate reader	450 nm	↑	Chen et al. (2022), Zarkovic et al. (2024)	Mouse liver, heart, A549 cells	Both
Colorimetric	MDA-TBA assay	MDA reacts with TBA to form red MDA-TBA adducts	Microplate reader	532 nm	↑	Li et al. (2023)	HepG2 cells, rat liver	Both
MS-based	LC-MS/MS	Detects oxidized phospholipids via high-resolution lipidomics	Agilent 6546 Q-TOF	Lipid profiling	↑	Kagan et al. (2017), Lin and Yang (2023), Sun et al. (2023), Xin et al. (2022)	HT1080, mouse liver	Both
MS-based	GC-MS	Detects volatile aldehydes (eg, 4-HNE) and oxidized fatty acid derivatives	Agilent 7890/5977 GC-MS	Aldehyde/fatty acid scan	↑	Lin and Yang (2023), Perez et al. (2020)	*Caenorhabditis elegans*, human cancer cells	Both

↑, increased signal upon lipid peroxidation; 4-HNE, 4-hydroxynonenal; MDA, malondialdehyde; ROS, reactive oxygen species.

#### Detection of Iron Accumulation

Iron released from the LIP drives lipid peroxidation via Fenton reaction and serves as a central mediator of ferroptosis (Chen et al., 2020). LIP levels can be visualized and quantified in live cells using iron–sensitive fluorescent probes such as FerroOrange, Calcein-AM, and Mito-FerroGreen. For fixed samples, Perl’s Prussian blue staining enables the detection of total and compartmentalized iron in tissues or cells. Iron chelators such as deferoxamine and deferiprone are commonly employed to functionally validate the iron dependence of ferroptosis. These agents bind Fe²⁺ and Fe³⁺, thereby reducing the LIP and rescuing cells from ferroptotic death in multiple experimental models (Entezari et al., 2022, Liang et al., 2024, Rayatpour et al., 2022). Collectively, these approaches provide both quantitative and functional evidence of the role of iron in ferroptosis. An overview of the commonly used methods for assessing iron accumulation in this context is provided in Table 3.

**Table 3:** Methods in measuring iron accumulation in cell or tissue

Method	Assay	Principle	Detection	λ/readout	Change	Reference	Cell/tissue	System
Colorimetric	Ferene assay	Fe²⁺ reacts with ferene to form a stable blue-colored complex	Microplate reader	593 nm	↑	Im et al. (2013), Liu et al. (2021)	HepG2 cells, liver tissue	Both
Fluorescent	Calcein-AM	Binds intracellular Fe²⁺, quenches green fluorescence	Fluorescence microscopy, flow cytometry	Ex/Em: 488/517 nm	↓	Kremer et al. (2021), Ma et al. (2015)	HT1080, pancreatic cancer cells	In vitro
Fluorescent	FerroOrange	Binds intracellular Fe²⁺ and emits orange fluorescence	Fluorescence microscopy, flow cytometry	Ex/Em: 543/580 nm	↑	Anandhan et al. (2023)	HEK293, neuronal cells	In vitro
Fluorescent	Mito-FerroGreen	Mitochondrial-selective probe emits fluorescence upon Fe²⁺ binding	Fluorescence microscopy	Ex/Em: 488/510 nm	↑	Tadokoro et al. (2023)	Cardiomyocytes, cancer cells	In vitro
Histochemical	Prussian blue	Fe³⁺ forms blue pigment with potassium ferrocyanide under acidic conditions	Light microscopy (DAB optional)	–	↑	Bitonto et al. (2023), Shen et al. (2022)	Mouse brain, liver tissue	In vivo

↑, increase in signal upon Fe²⁺/Fe³⁺ binding; ↓, decrease in signal upon Fe²⁺/Fe³⁺ binding.

### Molecular and Genetic Analyses

In addition to functional assays, molecular and genetic approaches are critical for validating ferroptosis and elucidating its underlying regulatory mechanisms. These strategies enable the quantitative assessment of ferroptosis-associated genes and proteins, providing insight into pathway activation and transcriptional responses. Loss- and gain-of-function experiments utilizing small interfering RNA, short hairpin RNA, CRISPR/Cas9, or transgenic overexpression facilitate the mechanistic dissection of key regulators implicated in ferroptotic signaling. Collectively, these methodologies complement biochemical observations and help to define the molecular context and upstream control of ferroptotic cell death. The representative approaches are summarized in Table 4.

**Table 4:** Representative molecular and genetic analyses in the context of ferroptosis

Category	Method	Application	Reference	Cell/tissue	System
Gene expression	qPCR	Iron metabolisms (TFRC, FTH1, SLC40A1, HMOX1)	Anandhan et al. (2023), Zhen et al. (2024)	HT1080, HepG2, mouse liver tissues	Both
		Lipid metabolisms (ACSL4, ALOX15, LPCAT3)	Cui et al. (2023), Ma et al. (2022)	A549, H1299, kidney/liver tissue	Both
		Antioxidant and glutathione metabolism (GPX4, SLC7A11, GCLC/GCLM, FSP1)	Koppula et al. (2022), Pan et al. (2022)	Lung cancer cell lines, xenograft tumor tissues	Both
		Ferroptosis-associated stress response markers (CHAC1, PTGS2)	Nghiem et al. (2025)	AML12, HK-2, mouse liver tissue	Both
	RNA-seq	Global transcriptomic profiling	Tran et al. (2023)	Human ischemic muscle, cancer cells	Both
Protein analysis	Western blot	Key ferroptotic regulators (GPX4, ACSL4, 4-HNE, MDA)	Cheng et al. (2020), Nghiem et al. (2025)	HT22, AML12, mouse liver tissues	Both
	ELISA	Quantitative protein detection in cell lysates or media (GPX4, MDA, 4-HNE)	Jia et al. (2020), Lin et al. (2022)	Mouse heart and liver tissues, HepG2	Both
	GPX activity assay	Enzymatic antioxidant capacity	Gaschler et al. (2018)	HeLa, mouse brain lysates	Both
Genetic tool	siRNA/shRNA	Knockdown of GPS4, SLC7A11 to study ferroptosis sensitivity	Li et al. (2025)	HepG2, A549, HT1080 cells	In vitro
	CRISPR/Cas9	Gene knockout (eg, GPX4, FSP1) to evaluate ferroptosis regulators	Koppula et al. (2022), Yang et al. (2023)	A549, H1299, GPX4 KO mice; FSP1 KO mice	Both
	Overexpression	Testing protective roles of GPX4, NRF2, p53, etc.	Lin et al. (2024)	Colorectal cancer cell lines	In vitro
Reporter assays	Luciferase	Transcriptional activity of NRF2 or p53	Anandhan et al. (2023), Wang et al. (2020)	HEK293, HepG2	In vitro

4-HNE, 4-hydroxynonenal; CHAC1, ChaC GSH–specific gamma-glutamylcyclotransferase 1; FSP1, ferroptosis suppressor protein 1; LPCAT3, lysophosphatidylcholine acyltransferase 3; shRNA, short hairpin RNA; siRNA, small interfering RNA; TFRC, transferrin receptor.

### Morphological Characterization

**Fig. 2:**
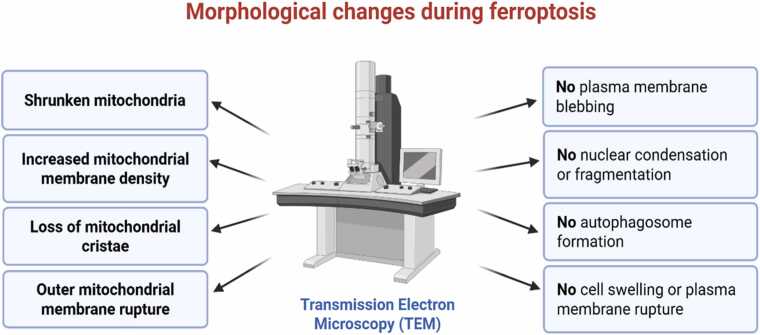
Morphological changes during ferroptosis as visualized by transmission electron microscopy (TEM). TEM reveals hallmark ultrastructural features of ferroptosis, including shrunken mitochondria with increased membrane density, loss of cristae, and occasional outer membrane rupture. Unlike apoptotic or necroptotic cells, ferroptotic cells retain intact nuclear structures and lack features such as chromatin condensation, membrane blebbing, plasma membrane rupture, or autophagosome formation.

Morphological analysis is essential to confirm ferroptosis and differentiate it from other forms of regulated cell death. Unlike apoptotic cells, which display nuclear fragmentation and membrane blebbing, and necrotic cells, which show plasma membrane rupture, ferroptotic cells do not exhibit these features. They do not form autophagosomes, as observed during autophagy. Instead, ferroptotic cells are characterized by distinct ultrastructural features such as condensed and shrunken mitochondria with increased membrane density, diminished or absent cristae, and intact outer mitochondrial membranes. Importantly, the nuclear morphology remains unaltered (Fig. 2) (Dixon et al., 2012). These features are most accurately observed using transmission electron microscopy (TEM), which remains the gold standard for ultrastructural analysis.

### Considerations and Pitfalls in Ferroptosis Detection

Although numerous tools have been developed to detect ferroptosis, challenges remain owing to its partial overlap with other cell death modalities. Lipid ROS accumulation or loss of viability alone is insufficient because similar features may occur during apoptosis or necrosis. Thus, robust identification requires a multimodal approach that combines biochemical markers (eg, lipid peroxidation, GPX4 loss), morphological changes (eg, mitochondrial shrinkage via TEM), and genetic or pharmacological validation with ferroptosis-specific inducers and inhibitors. Including negative controls for unrelated death pathways is essential to confirm specificity. Advanced biosensors, in vivo imaging, and ferroptosis-selective probes will enhance detection accuracy and biological insight.

### CONCLUDING REMARKS

Ferroptosis is a distinct, iron- and lipid peroxide-driven form of regulated cell death that is increasingly recognized in diverse disease contexts. Their mechanistic complexity necessitates the use of integrated detection strategies to ensure specificity. As the evidence for its role in pathology continues to grow, improved tools for real-time and in vivo detection are critical for translating basic insights into therapeutic advances. A better understanding of ferroptosis will deepen our knowledge of redox biology and open new avenues for targeted intervention.

## Author Contributions

**Fedho Kusuma:** Writing – review & editing, Writing – original draft. **Yeonsoo Joe:** Writing – review & editing. **Jaeseok Han:** Writing – review & editing, Writing – original draft. **Hun Taeg Chung:** Writing – review & editing, Writing – original draft. **Jeongmin Park:** Writing – review & editing. **Thu-Hang Thi Nghiem:** Writing – review & editing, Writing – original draft.

## Declaration of Competing Interests

The authors declare that they have no known competing financial interests or personal relationships that could have appeared to influence the work reported in this paper.
